# Human placenta-derived mesenchymal stem cells transplantation in patients with acute respiratory distress syndrome (ARDS) caused by COVID-19 (phase I clinical trial): safety profile assessment

**DOI:** 10.1186/s13287-022-02953-6

**Published:** 2022-07-28

**Authors:** Hamid Reza Aghayan, Fatemeh Salimian, Atefeh Abedini, Samrand Fattah Ghazi, Masud Yunesian, Sepideh Alavi-Moghadam, Jalil Makarem, Keivan Majidzadeh-A, Ali Hatamkhani, Maryam Moghri, Abbas Danesh, Mohammad Reza Haddad-Marandi, Hassan Sanati, Fereshteh Abbasvandi, Babak Arjmand, Pourya Azimi, Ardeshir Ghavamzadeh, Ramin Sarrami-Forooshani

**Affiliations:** 1 Cell Therapy and Regenerative Medicine Research Center, Endocrinology and Metabolism Molecular-Cellular Sciences Institute, Tehran University of Medical Sciences, Tehran, Iran; 2 ATMP Department, Breast Cancer Research Center, Motamed Cancer Institute, ACECR, P.O. BOX: 15179/64311, Tehran, Iran; 3 Chronic Respiratory Diseases Research Center, National Research Institute of Tuberculosis and Lung Diseases (NRITLD), Shahid Beheshti University of Medical Sciences, Tehran, Iran; 4 Department of Anesthesiology and Critical Care, Imam Khomeini Hospital Complex, Tehran University of Medical Sciences, Tehran, Iran; 5 Department of Environmental Health Engineering, School of Public Health, Tehran University of Medical Sciences, Tehran, Iran; 6 Genetics Department, Breast Cancer Research Center, Motamed Cancer Institute, ACECR, Tehran, Iran; 7 Scientific Research Center, Tehran University of Medical Sciences, Tehran, Iran; 8 Cancer and Cell Therapy Research Center, Tehran University of Medical Sciences, Tehran, Iran

**Keywords:** Acute respiratory distress syndrome (ARDS), Cell therapy, Coronavirus, COVID-19, Placenta mesenchymal stem cells (PL-MSC)

## Abstract

**Background:**

High morbidity and mortality rates of the COVID-19 pandemic have made it a global health priority. Acute respiratory distress syndrome (ARDS) is one of the most important causes of death in COVID-19 patients. Mesenchymal stem cells have been the subject of many clinical trials for the treatment of ARDS because of their immunomodulatory, anti-inflammatory, and regenerative potentials. The aim of this phase I clinical trial was the safety assessment of allogeneic placenta-derived mesenchymal stem cells (PL-MSCs) intravenous injection in patients with ARDS induced by COVID-19.

**Methods:**

We enrolled 20 patients suffering from ARDS caused by COVID-19 who had been admitted to the intensive care unit. PL-MSCs were isolated and propagated using a xeno-free/GMP compliant protocol. Each patient in the treatment group (*N* = 10) received standard treatment and a single dose of 1 × 10^6^ cells/kg PL-MSCs intravenously. The control groups (*N* = 10) only received the standard treatment. Clinical signs and laboratory tests were evaluated in all participants at the baseline and during 28 days follow-ups.

**Results:**

No adverse events were observed in the PL-MSC group. Mean length of hospitalization, serum oxygen saturation, and other clinical and laboratory parameters were not significantly different in the two groups (*p* > 0.05).

**Conclusion:**

Our results demonstrated that intravenous administration of PL-MSCs in patients with COVID-19 related ARDS is safe and feasible. Further studies whit higher cell doses and repeated injections are needed to evaluate the efficacy of this treatment modality.

*Trial registration*: Iranian Registry of Clinical Trials (IRCT); IRCT20200621047859N4. Registered 1 March 2021, https://en.irct.ir/trial/52947.

**Supplementary Information:**

The online version contains supplementary material available at 10.1186/s13287-022-02953-6.

## Background

In December 2019, severe acute respiratory distress syndrome was reported in patients with coronavirus 2 (SARA-CoV-2), a new strain of coronavirus, infection in Wuhan, China. This virus caused a pandemic disease called coronavirus disease 2019 (COVID-19) [1]. In contrast to most of the coronaviruses which cause mild disease in humans, SARS-CoV-2 and another closely related coronavirus, MERS-CoV, can cause lethal disease [2]. These two beta-coronaviruses have mortality rates of 10% and 37%, respectively [3]. COVID-19 primarily affects the respiratory system, but it is considered a multi-systemic disease and SARS-CoV-2 antigens have been detected in several organs [4]. Clinical manifestations vary from asymptomatic to severe multisystem disease [5]. The up-regulation of pro-inflammatory cytokines, coagulopathies, multi-organ dysfunction (MOD), and even death can occur in severe cases of COVID-19 infection [6]. Several studies have demonstrated the benefits of cell-based therapy in patients with acute respiratory distress syndrome (ARDS) [7]. In most of these studies, mesenchymal stem cells (MSCs) have been used and few of them have investigated pulmonary epithelial progenitor cells, endothelial progenitor cells, and pluripotent stem cells [8]. These studies have shown the role of MSCs in the reduction of inflammation, pulmonary tissue damage, and mortality in patients with ARDS [9]. MSCs are non-hematopoietic stem cells with regenerative, anti-inflammatory, and immunomodulatory properties [10]. These cells can be harvested from different sources such as bone marrow, adipose tissue, umbilical cord, cord blood, and placenta. MSCs from different tissues exhibit varying degrees of proliferation, differentiation, and immunomodulation. It has been demonstrated that fetal and perinatal‏‏ tissue-derived MSCs have more proliferative, anti-inflammatory, immunomodulatory, and differentiation capacity than their adult counterparts [11]. The anti-inflammatory and immunomodulatory properties of MSCs make them ideal candidates for the treatment of inflammatory lung diseases [12]. MSCs can inhibit B-lymphocytes, T-lymphocytes, and natural killer (NK) cells by secretion of anti-inflammatory cytokines like IL-10 and IL-4 [13]. It has been suggested that MSCs can prevent the activation of macrophages by reducing the expression of MHC-II, CD11, and CD83 [13]. They also reduce the secretion of pro-inflammatory cytokines like TNF-α, and IL-12 [14]. Cell–cell contact and secretion of soluble factors (cytokines) have been suggested as the main mechanisms of MSCs' anti-inflammatory and immunomodulatory activities. Surface markers like CD106 and CD54 have been considered important for immune modulation of MSCs through cell–cell contact. In comparison with bone marrow, adipose tissue, amniotic membrane, and umbilical cord-derived MSCs, PL-MSCs express a higher amount of CD106. MSCs secrete wide ranges of soluble factors among them Prostaglandin E2 (PGE2), Indoleamine 2,3-Dioxygenase 1(IDO1), transforming growth factor-beta 1 (TGF-b1), hepatocyte growth factor (HGF), Interleukin 6 (IL-6), HLA-G, Nitric oxide (NO)], and Galectin-1 plays a major role in their immunomodulatory activities [15, 16]. A large population of MSCs accumulates in the lungs after intravenous infusion, which can prevent pulmonary fibrosis, improve respiratory function, protect epithelial cells of the alveoli, and improve the pulmonary microenvironment [17]. Due to that the tissue origin and the culture process of MSCs have direct effects on their function, we designed the current phase I clinical trial to evaluate the safety of intravenous administration of PL-MSCs in patients suffering ARDS caused by COVID-19.

## Materials and methods

### Trial design and participants

**Fig. 1:**
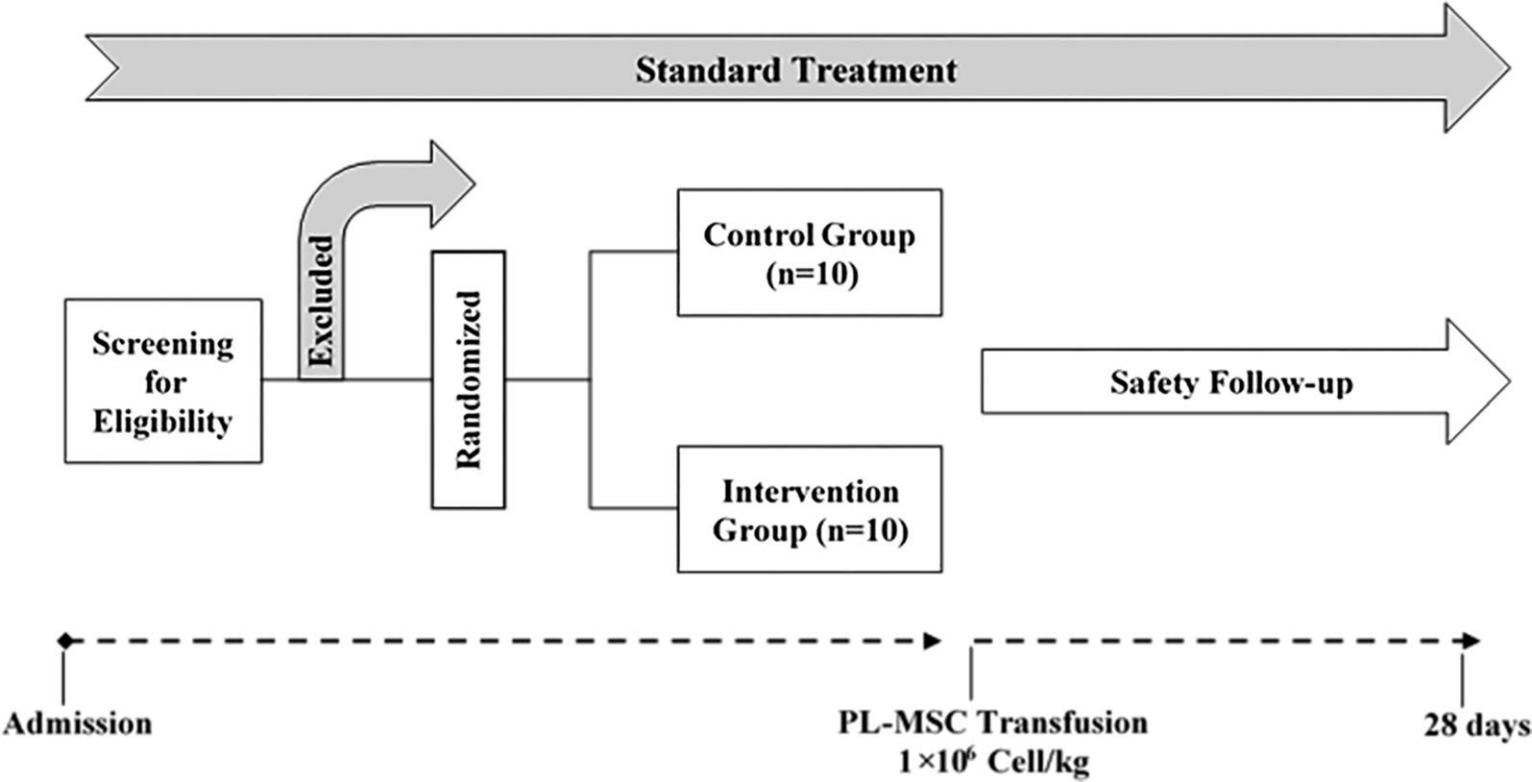
Clinical trial flow chart. Structure and patient enrollment in the trial

This non-blinded phase I study was designed to evaluate the safety of human PL-MSCs transplantation in patients with ARDS induced by COVID-19. Critically ill adult patients who were admitted to the ICU of two hospitals were considered as eligible for cell therapy according to the inclusion and exclusion criteria (Table 1). Patients were randomly divided into the treatment (*n* = 10) and control (*n* = 10) groups. The patients in the treatment group received a single injection of PL-MSCs (1 × 10^6^ cells/kg) through the intravenous cannula. The cell suspension was slowly injected (for 15 min), and the patient’s vital signs were continually monitored. Standard treatments were continued in both groups. Patients were evaluated daily for 28 days after transplantation until discharge or death. They were evaluated every 2 h in terms of vital signs (temperature, blood pressure, and heart rate) and every day in terms of laboratory parameters (biochemistry and hematology parameters) (Fig. 1). The project (including placenta donation and PL-MSCs manufacturing process) was approved by the Research Ethics Committees of Motamed Cancer Institute-Academic Center for Education, Culture, and Research (IR.ACECR.IRCRC.REC.1399.009). Before the injection process, written informed consent was obtained from conscious patients or next of kin in unconscious patients. The clinical trial protocol was registered in the Iranian Registry of Clinical Trials (IRCT20200621047859N4).

**Table 1:** Inclusion and exclusion criteria

Inclusion criteria	Exclusion criteria
≥ 18 years of age	< 18 years of age
The ability to understand and sign the informed consent (in case of unconsciousness consent is obtained from the next of kin)	History of chronic pulmonary disease with PaCO_2_ > 50 mmHg or history of using oxygen at home
Evidence of pneumonia by chest CT-scans and/or confirmation of SARS-CoV-2 by qRT-PCR	Pregnancy or breastfeeding
Bilateral opacity of the lungs on CT scan	History of pulmonary embolism or DVT in the past three months
Requires mechanical ventilation to increase oxygen saturation	History of lung transplantation
PaO_2_/FiO_2_ ratio ≤ 200	Existence of active malignancy that has been treated for the past two years
	More than 96 h have passed since the diagnosis of ARDS (the Berlin definition of ARDS)
	Moderate to severe liver failure (Childs-Pugh Score > 12)
	Extensive trauma in the last 5 days
	Existence of severe and irreversible disease with a probability of life expectancy of fewer than 6 months

### Isolation and expansion of PL-MSCs

Placenta samples were collected aseptically at the time of cesarean section delivery from normal full-term pregnancies. Written informed consents were obtained from all donors according to the national ethical guidelines for research on stem cells and regenerative medicine [18]. Donor’s medical records including physical examination and laboratory tests were reviewed and a blood sample was taken for complimentary laboratory tests. The presence of HIV, HBV, HCV, CMV, EBV, HTLV, Toxoplasma, and venereal diseases was evaluated by appropriate laboratory tests. PL-MSCs were manufactured according to our previously published protocol with few modifications [19]. To omit xenogeneic materials, fetal bovine serum (FBS) and collagenase NB6 (SERVA Electrophoresis, Germany) were substituted with human platelet lysate (hPL, PLBioscience, Germany) and animal-origin free collagenase CLSAFA/AF (Worthington, USA), respectively. Briefly, the fetal membrane was removed from the placenta and the chorionic plates were dissected out. The dissected tissues were washed thoroughly with CliniMACS PBS/EDTA buffer (MiltenyiBiotec, Germany) and minced into small fragments. Tissue fragments were digested by collagenase CLSAFA/AF at 37 °C for 90 min. To stop digestion, cold CliniMACS PBS/EDTA was added and the tissue digest passed through a 100 µm cell strainer (SPL, South Korea). After centrifugation (300 g/5 min), the cell pellets were resuspended in CliniMACS PBS/EDTA and layered on FicollPaque premium (GE Healthcare, USA). To isolate mononuclear cells (MNCs), centrifugation was performed at 500*g* for 20 min. The isolated MNCs were washed, resuspended in culture media (low glucose Dulbecco’s Modified Eagle Medium (DMEM-LG, Biowest, France), 5% hPL, and 2 Unit/ml injectable heparin), counted, seeded into 175 cm^2^ culture flasks (SPL, South Korea), and cultured in a CO_2_ incubator (37 °C, 5% CO_2_, and 95% humidity). After 72 h, non-adherent cells were discarded and fresh culture media was added to each flask. Media change was done twice a week and PL-MSCs were subcultured at 80 to 90% confluency using CTS™ TrypLE™ Select (Thermo Fisher Scientific, USA). Cell count and viability were assayed by hemocytometer using trypan blue dye. To characterize the PL-MSCs, CD marker expression (CD45, CD34, HLA-DR, CD105, CD73, and CD90) was analyzed by flow cytometry (Saba Biomedicals laboratory, Tehran, Iran). To evaluate their multilineage differentiation capacity, StemPro® Osteogenesis and Adipogenesis Differentiation Kits (Thermofisher, USA) were used according to the manufacturer's instructions.

### Cryopreservation of PL-MSCs

At the fifth subculture, PL-MSCs were harvested at 90% confluency, centrifuged (200 g/5 min), resuspended in cryopreservation media (DMEM-LG + 5% hPL + 10% DMSO (CryoMACS DMSO, MiltenyiBiotec, Germany)), and aliquoted into 2 ml cryovials (Corning, USA). Mr. Frosty container (Nalgene, Thermo Fisher Scientific, USA) was applied for slow freezing of samples according to the manufacturer’s instruction. Then, the cryopreserved PL-MSCs were transferred to the vapor phase of the liquid nitrogen tank (Statebourne Cryogenics, UK).

### Preparation of PL-MSCs for transplantation

Before transplantation, the cryopreserved PL-MSCs were rapidly thawed (at 37 °C water bath) and diluted in 0.9% injectable saline solution (IPH Co., Iran). To remove cryopreservation media and cell debris, the cells were washed twice (200 g/5 min). The cell pellet was resuspended in 25 ml of 0.9% injectable saline solution and passed through a 100 µm cell strainer (SPL, South Korea). Cell count and viability were analyzed by NucleoCounter® NC-100 device (Chemometec, Denmark). The cut-off point for cell viability was considered 70% and the required cell dose was adjusted to the number of viable cells. Approximately 1 × 10^6^ viable cells/kg were packed into a 50 ml CryoMACS® freezing bag (Miltenyi Biotec, Germany). The final product was transferred to a cool box containing ice packs and a data logger and then was shipped for transplantation. All processing steps were done under biosafety cabinets (Class A) in a Class B cleanroom (GMP facility, Cell Therapy and Regenerative Medicine Research Center, Tehran, Iran). In addition to cleanroom monitoring (temperature, humidity, differential pressure, microbial contamination, and airborne particle count), the final product was tested for microbial contamination, mycoplasma, gram staining, and endotoxins. To ensure chromosomal stability, the first and sixth subcultures of each lot were analyzed by G-band karyotyping (Pathology Department, Children Medical Center, Tehran, Iran).

### Outcome measurement

The main outcome of this phase I clinical trial was to determine the safety of intravenous PL-MSCs transplantation in patients with ARDS induced by COVID-19, who did not respond to the conventional therapies. To determine the safety of transplantation, early side effects including skin rash, changes in blood pressure, heart attack, changes in the respiratory capacity, and anaphylactic shock were evaluated continuously for 48 h. Vital signs and clinical parameters of the patients including temperature (T), pulse rate (PR), blood pressure (BP), respiratory rate (RR), and arterial oxygen saturation (O2SAT) were also evaluated until the day of discharge or death.

### Laboratory parameters measurement

Routine hematological and biochemical tests including white blood cell count(WBC), red blood cell count (RBC), Hematocrit (Hct), platelet count (Plt), hemoglobin level (Hb), neutrophil (Neutr) and lymphocyte (Lymph) percentage, blood urine nitrogen (BUN), creatinine (Cr), sodium (Na), and potassium (k) concentrations were performed. A coagulation panel including prothrombin time (PT) and partial thromboplastin time (PTT) was also evaluated. Changes in T lymphocyte subpopulation counts (CD4+, CD8+) were also measured on the day before and after injection in the treatment group.

### Statistical analysis

We presented the demographic data of the participants in Table 2 and considered delta (Δ) to reduce the effect of differences in baseline conditions. The data were analyzed using the IBM SPSS version 26.0 (Statistical Package for the Social Sciences, USA). Kolmogorov–Smirnov test was performed to test the normal distribution of the raw data. *T* test was used to analyze the differences seen in data with normal distribution and Mann–Whitney *U* was used for not normally distributed data. The tests were two-sided and a *P* value of < 0.05 was considered as a significant difference.

**Table 2:** Baseline characteristics of 20 enrolled patients with COVID-19

Patient ID	Intervention group (*n* = 10)									
	Survivors					Non-survivors				
	T1	T2	T5	T7	T10	T3	T4	T6	T8	T9
Age (years)	40–49	50–59	50–59	40–49	70–79	70–79	30–39	70–79	70–79	60–69
Gender	2	2	1	2	2	1	2	2	1	1
Duration of Hospitalization	10	80	6	9	6	11	4	9	15	16
Weight(kg)	80	95	92	100	87	78	110	73	75	81
Underlying disease	DM	–	DM, ILD, HTN	IBS	HTN	DM	Sarcoidosis	–	Asthma, HT, HTN	HTN, RA
Interval between ICU admission and cell injection (day)	2	1	1	5	2	3	3	2	12	4
Interval between cell injection and discharge/death (day)	6	78	4	3	3	5	1	6	0	11

Patient ID	Control group (*n* = 10)									
	Survivors					Non-survivors				
	C3	C5	C7	C9	C10	C1	C2	C4	C6	C8
Age (years)	80–89	70–79	70–79	60–69	30–39	30–39	50–59	60–69	50–59	70–79
Gender	2	1	1	2	2	2	2	2	2	2
Duration of Hospitalization	7	6	28	7	7	11	9	3	6	9
Weight(kg)	65	70	75	85	90	104	90	69	80	100
Underlying disease	**–**	DM, HTN	Sarcoidosis, asthma, Liver Hemangioma	**–**	**–**	**–**	CKD	DM, HTN	HTN,DM	DM, HTN
Interval between ICU admission and cell injection (day)	–	–	–	–	–	–	–	–	–	–
Interval between cell injection and discharge/death (day)	–	–	–	–	–	–	–	–	–	–

*DM* DiabetesMellitus, *ILD* Interstitial Lung Disease, *HTN* Hypertension, *IBS* Irritable Bowel Syndrome, *HT* Hypothyroidism, *RA* Rheumatoid Arthritis, *CKD* Chronic Kidney Disease

## Results

### Trial design

A total of 20 patients (age ≥ 18 years) who were admitted to the ICUs and had a positive test confirmed by PCR for SARS-CoV-2 with relevant clinical symptoms were enrolled in the study according to the inclusion criteria (Table 1). Ten were randomized to the PL-MSCs treatment group and ten to the control group (Fig. 1). Baseline characteristics of both groups were recorded. In this clinical trial, an attempt was made to match the individuals in the treatment and control groups in terms of age, weight, and concurrent medical conditions (Table 2). The mean age (control = 58.4, treatment = 62.3) and weight (control = 70.3, treatment = 58.1) were not significantly different (*P* ≤ 0.05).

### PL-MSCs manufacturing

**Fig. 2:**
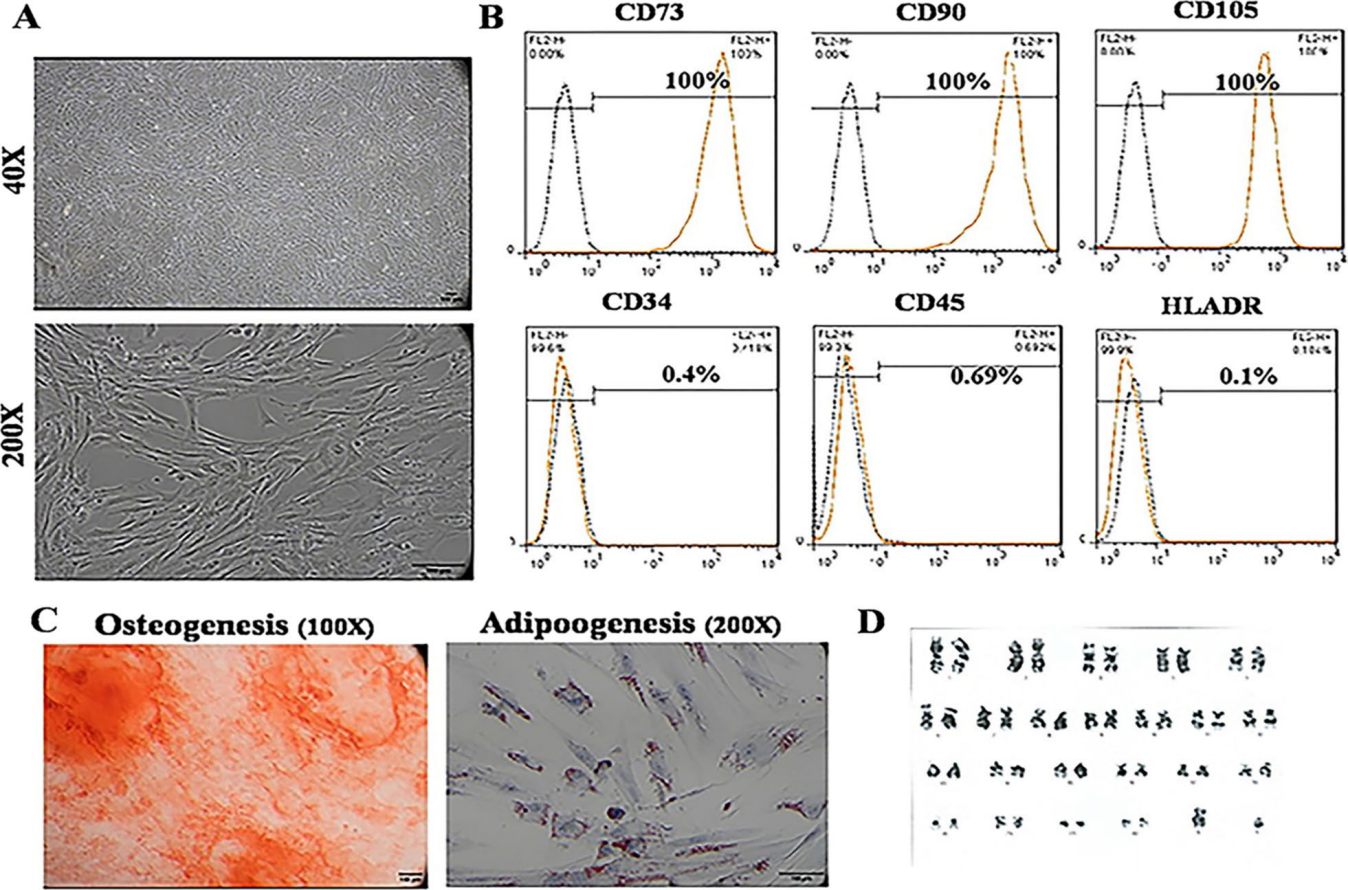
**A** PL-MSCs’ morphology under the inverted microscope with phase contrast, **B** CD markers expression pattern (the dotted black lines are isotype controls and the orange lines are the CD markers, **C** Differentiation of PL-MSCs into osteocytes (Alizarin Red staining) and adipocytes (Oil Red O staining), **D** results of karyotyping

GMP compliant PL-MSCs were successfully manufactured using our xeno-free protocol. Figure 2A demonstrates the spindle shape morphology of PL-MSCs at the fifth subculture. Flow cytometry analysis revealed that PL-MSCs express CD90, CD73, and CD105 and did not express CD45, CD34, and HLA-DR (Fig. 2B). They could differentiate into adipocytes and osteocytes after treatment with appropriate differentiation media (Fig. 2C). Figure 2D illustrates the karyotyping result of PL-MSCs at the sixth subculture. Post-thaw viability was more than 80% and the time between thawing and transplantation was less than 4 h.

### Safety and tolerability

**Fig. 3:**
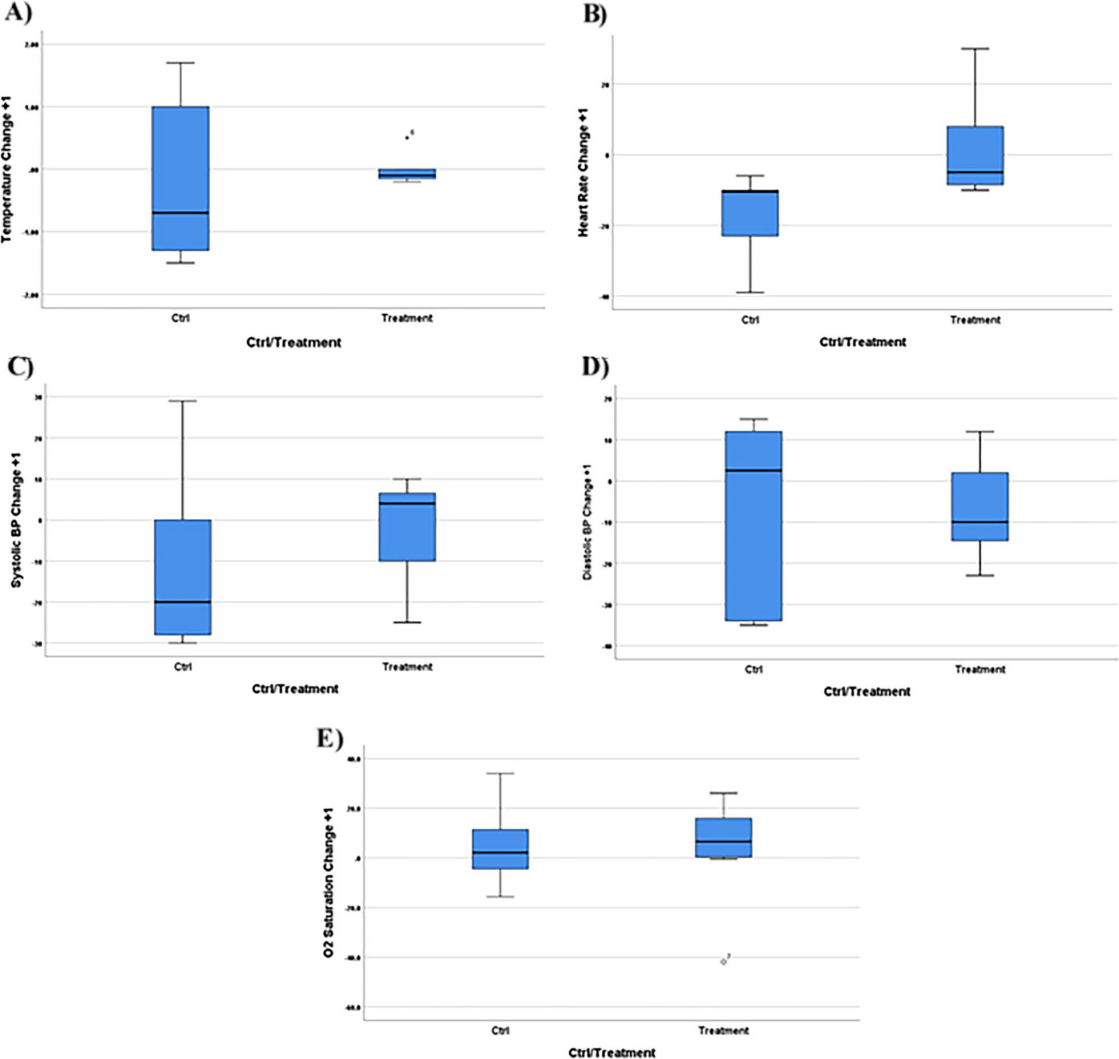
The changes in the vital signs 24 h after PL-MSC transplantation and comparison to the control group. Body temperature (**A**), heart rate (**B**), systolic blood pressure (**C**), and O_2_ saturation (**E**) were increased after PL-MSC transplantation but the diastolic blood pressure (D) was decreased compared to the control group. None of these differences were statistically significant

All patients were monitored for vital signs (T, BP, HR, and O2SAT) for 24 h after the transplantation. The treatment group did not show any adverse events related to the cell transplantation. A slight fever and shivering were observed after injection in patients T3 (a fever of 1 °C accompanied with shivering for 1 h) and T6 (a fever of 1.5 °C accompanied with shivering for 2 h) which were resolved spontaneously without any additional intervention (Fig. 3 and Additional file 1: Table S1).

### Clinical outcomes of the study groups

**Fig. 4:**
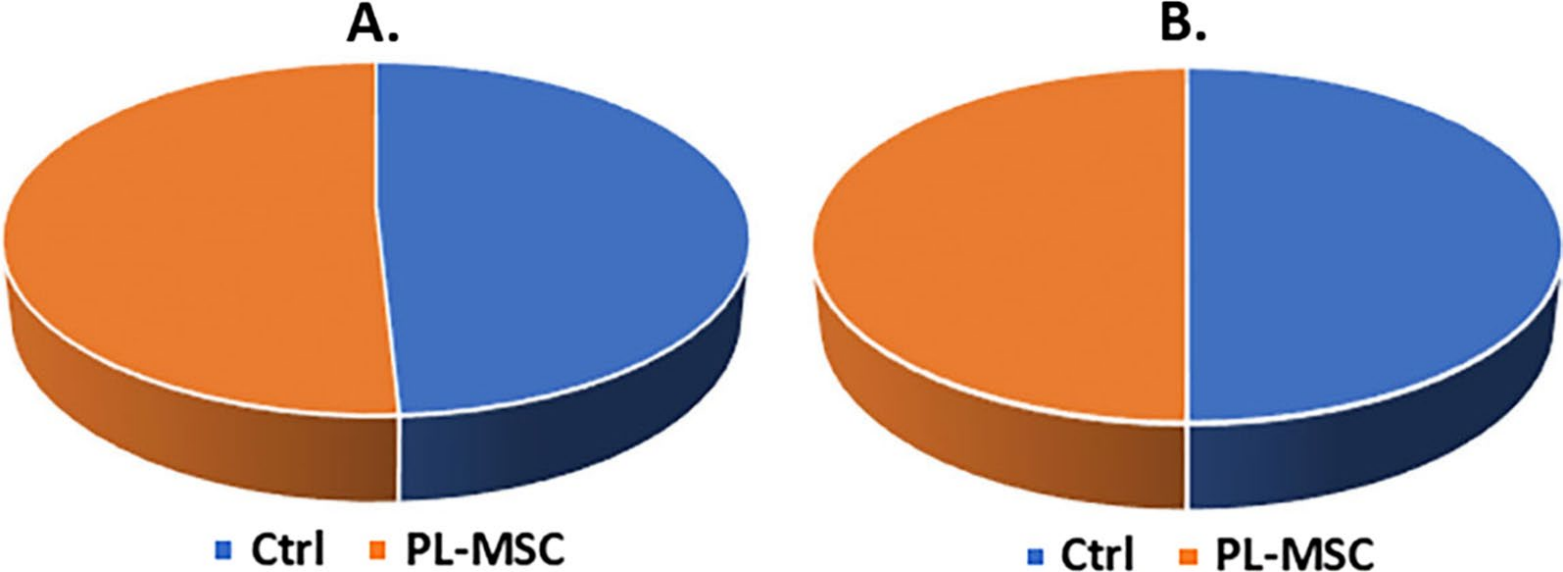
Duration of hospitalization (**A**), mortality (**B**)

**Fig. 5:**
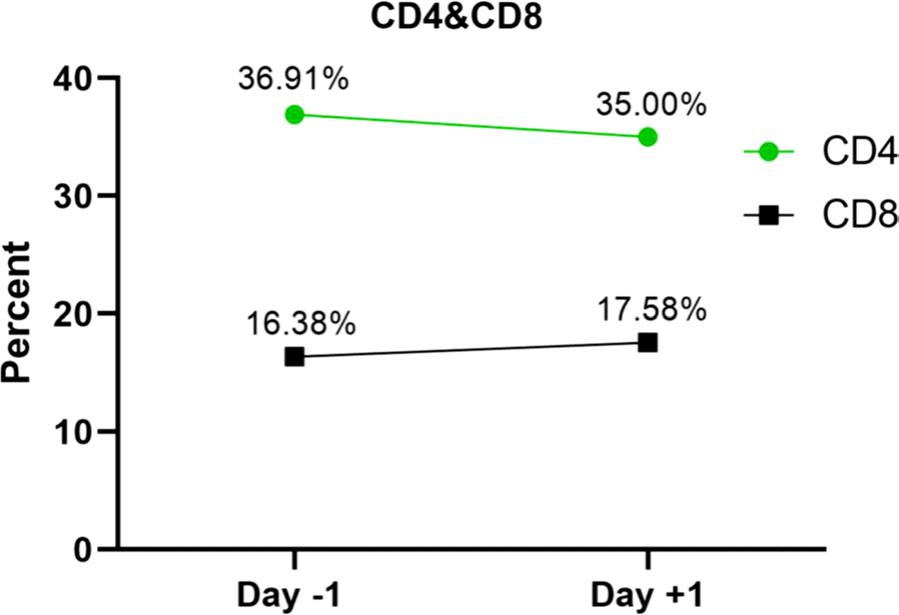
Alteration of CD4+ and CD8+ cells in patients with COVID-19 on day − 1 (before) and + 1 (after) PL-MSC transfusion were statistically insignificant (P ≥ 0.05)

Follow-up results demonstrated that the mortality rate in the treatment and control groups were equal (50%). Also, the duration of hospitalization of the recovered people was almost equal in both groups (Fig. 4A, B). Changes in the biochemistry and hematology parameters (Na, K, BUN, Cr, PT, PTT, WBC, RBC, Hb, Hct, Plt, Neutr, and Lymph) on days 1 and 3 in comparison to their baseline values (Δ + 1 and Δ + 3) were not statistically significant (Table 3). The average number of CD4+ and CD8+ T-cells did not change significantly 24 h after treatment (*P* ≥ 0.05) (Fig. 5).

**Table 3:** Laboratory parameters in the patients of intervention group and control group on days 1 and 3 in comparison to their baseline values (Δ + 1 and Δ + 3)

Patient ID	Intervention group (*n* = 10)									
	Survivors					Non-survivors				
	T1	T2	T5	T7	T10	T3	T4	T6	T8	T9
*Na*										
Δ + 1	− 2	1	1	1	− 1	2	5	− 2	NA	− 1
Δ + 3	− 7	0	0	1	− 3	0	NA	2	NA	− 3
*K*										
Δ + 1	− 0.2	0	0.1	0.3	− 0.1	− 0.4	− 0.4	− 0.4	NA	− 0.5
Δ + 3	0.4	0.2	− 0.2	− 0.2	0.3	− 0.5	NA	0.6	NA	− 1
*BUN*										
Δ + 1	18	NA	10	4	1	− 12	23	15	NA	4
Δ + 3	4	29	10	− 6	− 5	− 30	NA	67	NA	9
*Cr*										
Δ + 1	0	NA	0	0	11	8.3	0	4.7	NA	11.1
Δ + 3	0	9	0	− 10	0	− 33	NA	9.5	NA	0
*PT*										
Δ + 1	0	− 0.5	0.5	− 0.1	− 0.2	0.6	− 0.2	− 1	NA	1.5
Δ + 3	0	− 0.7	2.3	0	0.9	1.3	NA	− 2.2	NA	0.7
*PTT*										
Δ + 1	24	1	2	2	− 20	11	− 7	3	NA	0
Δ + 3	13	− 1	3	1	− 18	1	NA	4	NA	0
*WBC*										
Δ + 1	− 5	2.4	2.7	1.5	1.1	− 2.2	1.7	− 1.2	NA	− 7.1
Δ + 3	− 9.6	2.1	4	− 4.5	2.6	− 4.4	NA	4.6	NA	− 3.5
*RBC*										
Δ + 1	− 0.2	0.14	0.1	0.01	− 0.07	0.33	0.04	0.2	NA	0.05
Δ + 3	0.46	0.42	0.3	0.15	0.01	1.84	NA	− 0.2	NA	0.23
*Hb*										
Δ + 1	− 0.3	0.6	0.3	0.2	− 0.2	0.3	0.3	0.6	NA	0
Δ + 3	0.9	2.2	0.7	0.5	− 0.2	5	NA	− 0.9	NA	0.4
*Hct*										
Δ + 1	− 1.9	1.1	0.7	0.3	0	3.5	1.4	1.5	NA	0.2
Δ + 3	2.8	4.1	2.4	1.4	− 0.2	18.7	NA	− 1.4	NA	2.1
*Plt*										
Δ + 1	− 1	7	25	− 32	− 19	− 48	53	21	NA	2
Δ + 3	29	2	77	− 83	− 7	− 198	NA	− 44	NA	68
*Neutr*										
Δ + 1	− 2.1	3.5	− 1.2	4	0.3	4.6	− 0.4	3.6	NA	4.1
Δ + 3	− 5.8	4.6	0.5	7	4.6	4.3	NA	7.1	NA	− 2
*Lymph*										
Δ + 1	1.1	− 2.7	0.5	− 3	0	− 1.9	− 0.6	1.5	NA	0.1
Δ + 3	3.6	− 2.9	− 0.5	− 3.1	− 2.3	− 1.9	NA	− 3.8	NA	2

Patient ID	Control group (*n* = 10)									
	Survivors					Non-survivors				
	C3	C5	C7	C9	C10	C1	C2	C4	C6	C8
*Na*										
Δ + 1	5	− 1	− 1	6	0	2	2	− 1	3	− 6
Δ + 3	5	0	− 1	6	3	8	10	NA	NA	− 4
*K*										
Δ + 1	− 1.4	0.2	0.3	− 0.6	0.3	− 0.2	0.7	0	− 0.2	0.2
Δ + 3	− 1.2	0.1	− 0.3	− 1	0.4	− 0.7	0.2	NA	NA	0.6
*BUN*										
Δ + 1	NA	− 1	1	3	− 7	− 14	79	4	8	42
Δ + 3	NA	− 22	1	− 1	− 2	36	85	NA	− 12	31
*Cr*										
Δ + 1	0	− 21	0	− 8.3	0	0	50	18	− 7.1	54.5
Δ + 3	− 18	− 35	0	− 25	− 8.3	22	14	NA	− 28	45.4
*PT*										
Δ + 1	NA	− 0.4	0.8	NA	− 1.1	NA	0.3	NA	1.1	− 0.4
Δ + 3	NA	− 8	0.1	NA	0.2	NA	2.3	1.2	1.6	1.2
*PTT*										
Δ + 1	2	− 8	0	NA	− 3	NA	− 3	NA	0	− 6
Δ + 3	2	− 8	4	NA	2	NA	− 7	− 1	10	− 3
*WBC*										
Δ + 1	− 2.2	2.7	1.4	− 0.7	− 0.2	NA	− 0.3	0	− 1.1	− 2.5
Δ + 3	− 4.4	4	− 4.5	− 6.5	1.2	NA	− 0.6	− 3	6	− 0.08
*RBC*										
Δ + 1	NA	− 0.2	− 0.04	0.33	0.13	NA	0.25	− 0.25	− 0.15	− 0.36
Δ + 3	NA	− 0.3	0.04	− 0.31	0.24	NA	0.25	− 0.21	− 0.18	− 0.32
*Hb*										
Δ + 1	NA	− 1.4	− 0.1	0.8	0.1	NA	0.7	− 0.6	− 0.2	− 1.1
Δ + 3	NA	− 2.2	− 1	1.2	0.6	NA	1	− 0.6	− 0.8	− 1
*Hct*										
Δ + 1	NA	− 2.5	− 0.2	3	0.8	NA	3.6	− 0.5	− 1.9	− 3.2
Δ + 3	NA	− 3.6	0.4	− 1.6	2.1	NA	3.8	− 2.6	− 2.1	− 3.2
*Plt*										
Δ + 1	NA	− 48	12	92	− 11	NA	33	65	− 9	− 16
Δ + 3	NA	− 57	− 21	22	53	NA	51	42	184	101
*Neutr*										
Δ + 1	NA	8.3	3.4	− 1.7	2.9	NA	− 2.9	NA	NA	1.1
Δ + 3	NA	7.2	4.8	− 3.1	6.5	NA	1	NA	3.6	2.6
*Lymph*										
Δ + 1	NA	− 7.4	− 1.6	1.7	− 3.5	NA	0.4	− 1.2	NA	1.2
Δ + 3	NA	− 6.4	− 2.6	2.5	− 7.2	NA	− 0.5	9.6	− 4.5	− 0.8

*WBC* white blood cells, *RBC* red blood cells, *Hct* Hematocrit, *Plt* platelet, *Hb* hemoglobin level, *Neutr* neutrophil, *Lymph* lymphocyte, *BUN* blood urine nitrogen, *Cr* creatinine, *Na* sodium, *K, PT* potassium, prothrombin time, *PTT* partial thromboplastin time

## Discussion

High morbidity and mortality rates of the COVID-19 pandemic have made it a global health priority [20]. Recent studies have demonstrated that like Severe Acute Respiratory Syndrome (SARS) and avian influenza, in COVID-19 large amount of inflammatory cytokines are released into the body. This triggers a strong inflammatory reaction (cytokine storm) which eventually leads to ARDS [21]. Several vaccines have been approved for human use to induce protection and prevent severe complications. However, vaccine resistance due to mutated forms of the virus is a big concern. In addition to the development of more potent vaccines against a new variant of the virus, it is crucial to find new treatment approaches to eliminate ARDS complications and to improve the outcomes. Anti-inflammatory and immunomodulatory therapies thus may be effective in improving the outcomes. MSCs have been studied for many years in the treatment of various diseases including ADRS because of their immunomodulatory and anti-inflammatory properties and have had some promising results [22]. In this phase I clinical trial, we evaluated the safety of intravenous PL-MSCs transplantation in patients with ARDS caused by COVID-19. Our results demonstrated that the intravenous administration of a single dose of PL-MSCs was safe in these patients. No adverse events related to transplantation including significant changes in systolic blood pressure, anaphylaxis, hypersensitivity reactions, cardiovascular complications, and inflammatory enzymes were noted. Several clinical trials have evaluated the safety of MSCs transplantation in the treatment of patients with ARDS. These clinical trials indicate that the administration of MSCs in patients with ARDS is safe, which is consistent with the results of our study [7, 23–25]. Since the COVID-19 pandemic, approximately 46 clinical trials have been registered to evaluate the safety and efficacy of MSCs transplantation in ARDS caused by COVID-19 infection [20]. A study in China demonstrated that intravenous administration of 1 × 10^6^ cells/kg of MSCs is safe in patients with pneumonia caused by COVID-19 [26]. Another study in Spain indicated the safety of stem cells derived from adipose tissue in patients with COVID-19 pneumonia [27]. In a recent study, Adas et al. treated critically ill COVID-19 patients with Wharton Jelly-derived MSCs and measured inflammatory cytokines. They did not detect any adverse events and observed a decrease in pro-inflammatory cytokines and an increase in anti-inflammatory cytokines that may play a role in regulating the cytokine storm in COVID-19 patients [28]. Another case report describes the safe and effective treatment of a critically ill COVID-19 patient with three doses of bone marrow MSCs [29]. Shu et al. reported that infusion of human umbilical cord mesenchymal stem cells in COVID-19 patients is safe. They recorded a rapid reduction in the levels of inflammatory factors, including IL-6 and CRP, and also a faster return of the lymphocyte count to the normal values [30]. In vitro studies have demonstrated that PL-MSCs have more immunomodulatory activities than other fetal-derived MSCs. It has shown that PL-MSCs act at Angiotensin-converting enzyme 2 (ACE-2) and Transmembrane protease, serine 2 (TMPRSS-2) receptors level and block further entry of the viral particles into pulmonary alveolar cells [31]. Besides immunomodulatory effects, MSCs can restore the capillary barrier, increase the concentration of alveolar ATP, and inhibit bacterial growth by secretion of the antimicrobial agent which reduce ARDS severity in the lungs [32]. T-cells are one of the most important components of the immune system against viral disease [33]. Studies have shown that the number of CD4+ and CD8+ T-cells is reduced in patients with severe COVID-19 infections [34]. In our study, there were no significant changes in the number of CD4+ and CD8+ cells, 24 h after cell transplantation. In the current study, we could not prove the efficacy of PL-MSCs transplantation in COVID-19 related ARDS. The primary endpoint of the current phase I clinical trial was the assessment of PL-MSCs transplantation safety and tolerability. To address this important issue and according to our IRB recommendation, we enrolled critically ill patients in the last stages of COVID-19 related pneumonia. Furthermore, we administered a single injection of low-dose PL-MSCs to all participants. Previous studies have shown the importance of timing in the therapeutic approach of the different stages of COVID-19 and demonstrated that aggressive anti-inflammatory treatment should be initiated at the beginning of cytokine storm [35]. The early inflammatory phase of COVID-19 has been suggested as the best time for immunomodulatory therapies [36]. In recent studies with promising results in COVID-19 related ARDS, multiple injections (mostly three injections) of higher doses of MSCs have been administered [37–39]*.* Another limitation of the current study was the small number of patients that may lead to the low statistical power.

## Conclusion

In conclusion, our findings suggest that intravenous administration of PL-MSCs is safe in patients with ARDS caused by COVID-19. Multiple injections of higher doses of PL-MSCs in the early inflammatory phase may improve its efficacy as a novel treatment in COVID-19 pneumonia.

## Supplementary Information

**Additional file 1: Table S1**. Vital signs of 20 enrolled patients with COVID-19 beforeintervention (0) and 1 day after intervention (+1).

## Data Availability

The data that support the findings of this study will be made available upon reasonable request to the corresponding authors.

## Abbreviations

ARDS: Acute respiratory distress syndrome
BP: Blood pressure
BUN: Blood urine nitrogen
COVID-19: Coronavirus disease 2019
Cr: Creatinine
DMEM-LG: Low glucose Dulbecco’s Modified Eagle Medium
FBS: Fetal bovine sera
Hb: Hemoglobin
Hct: Hematocrit
hPL: Human platelet lysate
ICU: Intensive care unit
IRB: Institutional review board
K: Potassium
Lymph: Lymphocyte
MNCs: Mononuclear cells
MOD: Multi-organ dysfunction
MSCs: Mesenchymal stem cells
Na: Sodium
Neutr: Neutrophil
NK: Natural killer
O2SAT: Arterial oxygen saturation
PL-MSCs: Placenta-derived mesenchymal stem cells
Plt: Platelet
PR: Pulse rate
PT: Prothrombin time
PTT: Partial thromboplastin time
RBC: Red blood cells
RR: Respiratory rate
T: Temperature
WBC: White blood cells
