# Supplementary material for: Human placenta-derived mesenchymal stem cells transplantation in patients with acute respiratory distress syndrome (ARDS) caused by COVID-19 (phase I clinical trial): safety profile assessment
Source: Stem Cell Res Ther. 2022 Jul 28;13:365. doi: 10.1186/s13287-022-02953-6 (PMC9330663; doi:10.1186/s13287-022-02953-6)
Supplement: Supplementary file 1 — Additional file 1: Table S1. Vital signs of 20 enrolled patients with COVID-19 beforeintervention (0) and 1 day after intervention (+1). [file 13287_2022_2953_MOESM1_ESM.docx]

**Table S1.** Vital signs of 20 enrolled patients with COVID-19 before intervention (0) and 1 day after intervention (+1).

|  |  |  | intervention group (n=10) | | | | | | | | | | Control group (n=10) | | | | | | | | | |
| --- | --- | --- | --- | --- | --- | --- | --- | --- | --- | --- | --- | --- | --- | --- | --- | --- | --- | --- | --- | --- | --- | --- |
|  |  | | **Survivors** | | | | | **Non-Survivors** | | | | | **Survivors** | | | | | **Non-Survivors** | | | | |
|  | **Patient ID** | | **T1** | **T2** | **T5** | **T7** | **T10** | **T3** | **T4** | **T6** | **T8** | **T9** | **C3** | **C5** | **C7** | **C9** | **C10** | **C1** | **C2** | **C4** | **C6** | **C8** |
| Temp, °C | **0** |  | 37 | 36.7 | 36.9 | 36.5 | 36.6 | 36.8 | 37 | 37 | 36.5 | 37 | 37.4 | 38 | 37.7 | 37.1 | 37 | 36.1 | 38.6 | 38.3 | 37 | 36.5 |
|  | **+1** |  | 36.8 | 36.6 | 36.8 | 36.5 | 36.7 | 36.8 | 36.8 | 37.5 | 36.5 | 36.9 | 36.5 | 37.4 | 37 | 36.4 | 36.5 | 37.8 | 37.1 | 37 | 36.5 | 37.5 |
| HR, beats/min | **0** |  | 81 | 71 | 75 | 80 | 92 | 55 | 87 | 105 | 90 | 70 | 109 | 99 | 97 | 88 | 86 | 120 | 106 | 93 | 90 | 73 |
|  | **+1** |  | 74 | 101 | 65 | 70 | 90 | 73 | 85 | 100 | 88 | 80 | 98 | 86 | 80 | 68 | 70 | 81 | 96 | 83 | 84 | 50 |
| Sys BP, mmHg | **0** |  | 120 | 134 | 144 | 124 | 128 | 131 | 108 | 145 | 151 | 135 | 142 | 135 | 141 | 128 | 122 | 145 | 96 | 141 | 139 | 150 |
|  | **+1** |  | 130 | 138 | 133 | 115 | 131 | 136 | 116 | 120 | 147 | 129 | 123 | 118 | 131 | 121 | 110 | 117 | 125 | 120 | 139 | 120 |
| Dias BP, mmHg | **0** |  | 74 | 82 | 92 | 83 | 81 | 94 | 70 | 81 | 91 | 92 | 111 | 95 | 73 | 91 | 88 | 105 | 64 | 65 | 79 | 90 |
|  | **+1** |  | 70 | 94 | 79 | 67 | 75 | 71 | 78 | 71 | 85 | 85 | 76 | 87 | 74 | 84 | 78 | 71 | 76 | 80 | 85 | 89 |

Temp; Temperature, HR; Heart Rate, Sys BP; Systolic Blood Pressure, Dias BP; Diastolic Blood Pressure
